# Prescribing Practices of Intravenous Immunoglobulin in Tertiary Care Hospitals in Malaysia: A Need for a National Guideline for Immunoglobulin Use

**DOI:** 10.3389/fphar.2022.879287

**Published:** 2022-06-09

**Authors:** Jian Lynn Lee, Shamin Mohd Saffian, Mohd Makmor-Bakry, Farida Islahudin, Hamidah Alias, Adli Ali, Noraida Mohamed Shah

**Affiliations:** ^1^ Centre for Quality Management of Medicines, Faculty of Pharmacy, Universiti Kebangsaan Malaysia, Kuala Lumpur, Malaysia; ^2^ Department of Pharmacy, Tengku Ampuan Rahimah Hospital, Ministry of Health Malaysia, Selangor Darul Ehsan, Malaysia; ^3^ Department of Pediatrics, UKM Medical Centre, Faculty of Medicine, Universiti Kebangsaan Malaysia, Kuala Lumpur, Malaysia

**Keywords:** prescribing practices, drug utilization, off-label use, intravenous immunoglobulin (IVIg), Malaysia

## Abstract

Rational use of drug involves the use of medicine as per clinical guidelines. Given the steady increase in the clinical utility of intravenous immunoglobulin (IVIG) either as licensed or off-label use, concerns are being raised about the possibility of supply shortages that could significantly impact patient care. Therefore, there is a need to regulate and to promote the rational use of this valuable medication. This cross-sectional chart review study attempts to evaluate the prescribing patterns of IVIG at two tertiary hospitals in Malaysia. Patients’ medical files and dispensing records were examined and compared with current guidelines. A total of 348 prescriptions for IVIG were written during the 1-year study period. The highest usage of IVIG was for neurological (47.9%), immunological (27.5%), and hematological conditions (20%). The number of prescriptions with the US Food and Drug Administration (FDA) licensed indications and off-label indications was 148 (42.5%) and 200 (57.5%), respectively. Age (OR: 1.02, 95% CI: 1.01–1.03, *p* = 0.003) and those admitted to the critical care units (OR: 11.11, 95% CI: 5.60–22.05, *p* < 0.001) were significant factors for receiving IVIG for an off-label indication. Most prescriptions (79%) had appropriate dosing. Significant factors associated with receiving inappropriate dose of IVIG include age (OR: 0.93, 95% CI: 0.89–0.97, *p* = 0.001) and those admitted to the critical care units (OR: 10.15, 95% CI: 3.81–27.06, *p* < 0.001). This study advocates the development and implementation of evidence-based clinical guidelines with prioritization protocol to ensure rational use of IVIG.

## Introduction

Immunoglobulins (IgG) are fractionated blood products derived from pooled human plasma of over a thousand healthy blood donors. The end products are composed of mainly immunoglobulin G (IgG) molecules (>90%), traces of other immunoglobulins, and excipients such as stabilizers and sodium (Abolhassani et al., 2015). The FDA has approved various preparations of immunoglobulin which can be administered either through intravenous, subcutaneous, or intramuscular. The intramuscular (IM) administration of IgG is uncommon, and the only product available in the market that can be given as IM is GamaSTAN, that is used as post-exposure prophylaxis for measles, hepatitis A, varicella, and rubella. The intravenous and subcutaneous formulation is usually given as IgG replacement, anti-inflammatory and immunomodulatory therapy. Although the subcutaneous is gaining popularity, the intravenous route of administering IgG is still the most common mode of IgG replacement therapy (Abolhassani et al., 2015).

The intravenous immunoglobulin (IVIG) was initially indicated for treating primary immunodeficiencies (PI) (Bruton, 1952; Abolhassani et al., 2015). Upon recognizing the anti-inflammatory and immunoregulatory properties of IgG, it has become an important treatment option for a range of autoimmune and acute inflammatory diseases with various success levels (Perez et al., 2017). There are presently more than 150 off-label uses of IVIG (Leong et al., 2008) and the usage is expected to continue growing. Common examples of off-label uses of IVIG which are supported by other guidelines but not FDA approved include neurological conditions such as Guillain-Barre Syndrome and myasthenia gravis as well as dermatological conditions such as toxic epidermal necrolysis (Department of Health, 2011; Jurisdictional Blood Committee, 2012; Prairie Collaborative, 2018). Despite insufficient evidence, the use of IVIG for off-label indications is commonly practiced by physicians worldwide (Dawoud et al., 2012; Tonkovic and Rutishauser, 2014; Shemer et al., 2018; Rezaie et al., 2019).

The supply of IVIG is limited because the products are derived from human plasma. Malaysia has a national policy for self-sufficiency in blood-derived therapies which is based on voluntary non-remunerated blood donation. Annually, approximately 80,000 packs of blood are sent to the Commonwealth Serum Laboratory (CSL), Australia for fractionation under the national fractionation contract (Burnouf, 2011; Isahak, 2021). However, the collection of immunoglobulin G is insufficient, thus most of the IVIG products are obtain from the global commercial market (Burnouf, 2011; Isahak, 2021). Scarcity remains the major concern regarding the increasing usage of IVIG for off-label indications, unlike the usual concern of drug safety in off-label use of medicine. For instance, a 9-year retrospective study conducted in Israel from 2007 to 2015 showed increased rates of 185 and 417% in the total number of patients treated with IVIG and the amount (in grams) of IVIG administered, respectively (Shemer et al., 2018). In Taiwan, a 10-years population study from 2008 to 2017 reflected an average of 10% per year increase in the distribution of IVIG (Hsu et al., 2021). Major worldwide shortages were noted in the late 1990s, whereby the demand for IVIG exceeded the supply by 20% in 1997 and by 30% in 1998 (Boulis et al., 2002). These events have impacted patients negatively, especially those who need it the most. In 2019, the United States was again faced with insufficient supply of IVIG, resulting in local shortages and rationing (HartmannGoogle, 2020). Additional tension on global plasma supply was introduced by the coronavirus disease 2019 (COVID-19) pandemic. The unprecedented global health crisis has not only resulted in a reduction in donor pool (Hartmann and Klein, 2020), but the demand has also increased due to the use of IVIG as an off-label adjunctive treatment for COVID-19 infection (Herth et al., 2020; Nguyen et al., 2020; Shao et al., 2020) and vaccine-induced thrombotic thrombocytopenia (VITT) caused by adenovirus-vectored COVID-19 vaccines (Bourguignon et al., 2021; Guetl et al., 2021; Karnam et al., 2021; Thaler et al., 2021).

Two formulations of the IgG approved for use in Malaysia are the intravenous immunoglobulin (IVIG) and the subcutaneous immunoglobulin (SCIG). However, the IVIG is the most common formulation used. Hospitals in Malaysia have started using IVIG in 1986 for the treatment of PI (Lokman et al., 1988). Since then, the demand has increased over the years because it is used as a primary or adjunct treatment in various conditions (Toh et al., 2014). Unlike countries such as Australia, Canada, and the United Kingdom which have clear prescribing guidelines and priority protocols for the supply of IVIG (Department of Health, 2011; Jurisdictional Blood Committee, 2012; Prairie Collaborative, 2018), most Malaysian hospitals do not have specific guidelines for IVIG use. The Malaysian Ministry of Health Medicines Formulary briefly states the approved indications and doses (Program Perkhidmatan Farmasi, 2021). Therefore, IVIG could potentially be overused for indications that are not well supported by recent evidence. This practice will lead to an increase in unnecessary healthcare costs to the country. Due to the increasing demand, limited availability, inadequate funding, and frequent interruptions of global plasma supply (Ruiz-Antorán et al., 2010), health care institutions are strongly advised to monitor the usage of IVIG and to reserve supplies for those with the utmost clinical need. Therefore, this study aims to evaluate the prescribing practices of IVIG in order to propose suitable approaches to regulate and to advocate its rational use in Malaysia.

## Methods

### Study Design and Setting

A prospective chart review study was conducted at two government tertiary care hospitals in Malaysia: Serdang Hospital and Universiti Kebangsaan Malaysia (UKM) Medical Center. Serdang Hospital is a 620-bedded hospital, while UKM Medical Center is a 1054-bedded university hospital. Both hospitals offer an array of specialized services in the field of medicine and surgery and also serve as referral centers in the country.

### Data Collection

All patients who received any IVIG products between 1st January 2019 and 31st December 2019 were included in the study. The list of patients, prescribers, formulation used, and quantity supplied were identified from the hospital’s pharmacy medication indenting system. The price of IVIG used was supplied by the pharmacy logistic department of the respective hospitals. Patients’ demographics, indications of IVIG, dosage regimen, and adverse drug reactions were gathered from patients’ medical records. IVIG usage was reviewed as separate prescriptions for patients who received IVIG multiple times during the study period.

### Categorization of Age, Ethnicity, and Medical Conditions

Patients were categorized according to the age group adapted from the Center for Drug Evaluation and Research, Food and Drug Administration (FDA) (Center for Drug Evaluation and Research FDA, 2014) and National Institute of Child Health and Human Development (Williams et al., 2012). Categorization of ethnicity was adapted from the Department of Statistics Malaysia while ethnicity was self-reported either by patients or their parents and documented in the medical records. Ethnicity data were collected to reflect the patients’ diversity in Malaysian government hospitals and to allow assessment for differences in prescribing practices. Medical conditions were classified according to the International Statistical Classification of Diseases and Related Health Problems (11th ed.; ICD-11) developed by the World Health Organization (2020) (WHO. International statistical classification of, 2020).

### Categorization of Licensed and Off-Label Indications

To evaluate the usage of IVIG according to the evidence-based prescribing, the prescribed indications were divided into licensed or off-label indications. Presently, licensed indications refer to the eight indications approved by FDA: 1) treatment of primary humoral immunodeficiency, 2) maintenance therapy to improve muscle strength and disability in adult patients with multifocal motor neuropathy (MMN), 3) prevention of bacterial infections in patients with hypogammaglobulinemia and/or recurrent infections associated with B- cell chronic lymphocytic leukemia, 4) prevention of infections, interstitial pneumonia, and acute graft-versus-host disease (GVHD) after bone marrow transplantation (BMT), 5) to increase platelet count rapidly in order to prevent and/or control bleeding in immune thrombocytopenia (ITP) or to allow patients with ITP to undergo surgery, 6) prevention of coronary artery aneurysm associated with Kawasaki disease, 7) treatment of chronic inflammatory demyelinating polyneuropathy (CIDP) to improve neuromuscular disability and impairment, as well as for maintenance therapy, and 8) to prevent relapse and to decrease the frequency of serious and minor bacterial infections in pediatric patients with human immunodeficiency virus (HIV) infection (Perez et al., 2017). Uses in conditions other than the approved indications were categorized as off-label indications.

### Categorization of Evidence and Basis of Recommendation

Level of evidence was classified as Ia, Ib, IIa, IIb, III, and IV; and strength of recommendation as A, B, C, and D. Definition of the categorization and strength of recommendation is available in Supplementary Table S1. The indications were then sub-categorized into an ordinal evidence category in clinical decision making regarding the benefit of treatment with IVIG, which comprised of ‘definitely beneficial’, ‘probably beneficial’, ‘may provide benefit’ or ‘unlikely to provide benefit’ (Perez et al., 2017). The categorization of evidence and basis of recommendation was determined according to the work group report of the American Academy of Allergy, Asthma and Immunology (Perez et al., 2017). This report is an updated comprehensive evidence-based guideline on immunoglobulin therapy use in human diseases. The basis for FDA-approved indications was reviewed while discussing other disease states in which immunoglobulin therapy has been applied.

### Definition of Inappropriate Dosing of Intravenous Immunoglobulin

Inappropriate dosing was defined as a deviation from the recommended dose by 20% (Kaji et al., 2006; Kaufmann et al., 2018), either under-dosing or over-dosing. The appropriateness of the prescribed IVIG dosages was compared using the product leaflets (Behring, 2016; Instituto Grifols, 2017) and clinical practice guidelines for IVIG use in Australia (Jurisdictional Blood Committee, 2012), Canada (Prairie Collaborative, 2018), and United Kingdom (Department of Health, 2008; Department of Health, 2011; NHS England Immunoglobulin Policy Working Group, 2019). These guidelines were chosen because of their comprehensiveness, recognition by several institutions, and widespread usage as references in other prescribing patterns studies (Torbic et al., 2021). Administered doses for each indication were compared with the recommended doses.

### Statistical Analysis

All information obtained was analyzed using the Statistical Package for Social Science (SPSS) software Version 26 (IBM Corp., Armonk, N.Y., United States). Normality test was performed using the Kolmogorov-Smirnov test. Continuous data were presented in mean and standard deviation (SD) if normally distributed, while non-normally distributed data were summarized using the median and interquartile range (IQR). Categorical data were reported descriptively in frequencies and percentages. The Pearson’s Chi-square test was used in comparing the categorical variables between two groups. On the other hand, continuous data between two groups were compared using either independent *t*-test or Mann-Whitney *U* test if data is normally distributed or not normally distributed, respectively.

Binary logistic regression analysis using the backward stepwise (likelihood ratio) method was performed to predict the odds of a patient receiving IVIG prescription for off-label indications and receiving inappropriate dosing of IVIG. Plausible variables affecting the physicians’ prescribing behavior included patients’ sex, age, ethnicity, and ward setting (Fakhari et al., 2018). Significance level was set at *p*-value < 0.05.

### Ethics Approval

This study was performed in accordance with the Declaration of Helsinki and was approved by the Universiti Kebangsaan Malaysia Research Ethics Committee (UKM PPI/111/18/JEP-2018–307) and the Medical Research and Ethics Committee, Ministry of Health Malaysia (KKM/NIHSEC/P18-1216 (10)). For this study which was based solely on routinely collected data, informed consent was waived by both ethics committees.

## Results

A total of 348 prescriptions were given to 240 patients, with 31 of them receiving more than one course of IVIG treatment during the 1-year period. Patients who repeated the course of IVIG were for the treatment of PI (*n* = 7), ITP (*n* = 6), post-operative sepsis in children (*n* = 5), sepsis in children (*n* = 3), Kawasaki disease (*n* = 2), GBS (*n* = 2), SLE (*n* = 2), myasthenia gravis in crisis (*n* = 2), and autoimmune anti-NMDA encephalitis (*n* = 2). Patients’ age was not normally distributed with a median (Interquartile range, Q1 to Q3) of 5.08 years (2 months–46 years old) and ranging from 1 day old to 76 years old. Two types of IVIG preparations were used, 2.5g/50 ml (5%) and 3.0g/50 ml (6%). No adverse drug reaction was documented in this study. The total expenditure of IVIG for the year 2019 for both hospitals was MYR 3,698,529 (885,218 USD) for 15,652.5 g used. This gives an average of 50 g of IVIG used per prescription. The summarized data of included prescriptions is presented in Table 1.

**TABLE 1:** Summarized data of included prescriptions (*n* = 348).

Parameter	
Sex, n (%)	
Male	184 (52.9)
Female	164 (47.1)
Median age (IQR; min, max)	5.08 (2 months - 46 years; 1 day, 76 years)
Age class, n (%)	
Preterm neonates (<37-weeks PMA up to 1 month)	21 (6.0)
Term neonates (≥37-weeks PMA up to 1 month)	55 (15.8)
Infants (1 month up to 2 years)	61 (17.5)
Children (2 years up to 12 years)	57 (16.4)
Adolescents (12–18 years)	20 (5.7)
Adult (≥18 years)	134 (38.5)
Ethnic group, n (%)	
Bumiputera^a^	264 (75.9)
Chinese	68 (19.5)
Indians	8 (2.3)
Others^b^	8 (2.3)
Ward settings, n (%)	
Critical care units	155 (44.5)
Daycare units	71 (20.4)
General wards	122 (35.1)
Total grams of IVIG used, grams	15,652.5
2.5 g/50 ml bottle, grams	10,537.5
3.0 g/50 ml bottle, grams	5115.0
Total cost^c^	MYR 3,698,529 (885,218 USD)

PMA, post-menstrual age; IVIG, intravenous immunoglobulin; MYR, malaysian ringgit.

a Bumiputera includes Malays (*n* = 260) and the natives from Sabah (*n* = 3) and Sarawak (*n* = 1).

b Others include non-Malaysian citizens.

c Total cost calculation was based on the mean cost of IVIG, of MYR236.29 (57 USD) per Gram.

**FIGURE 1:**
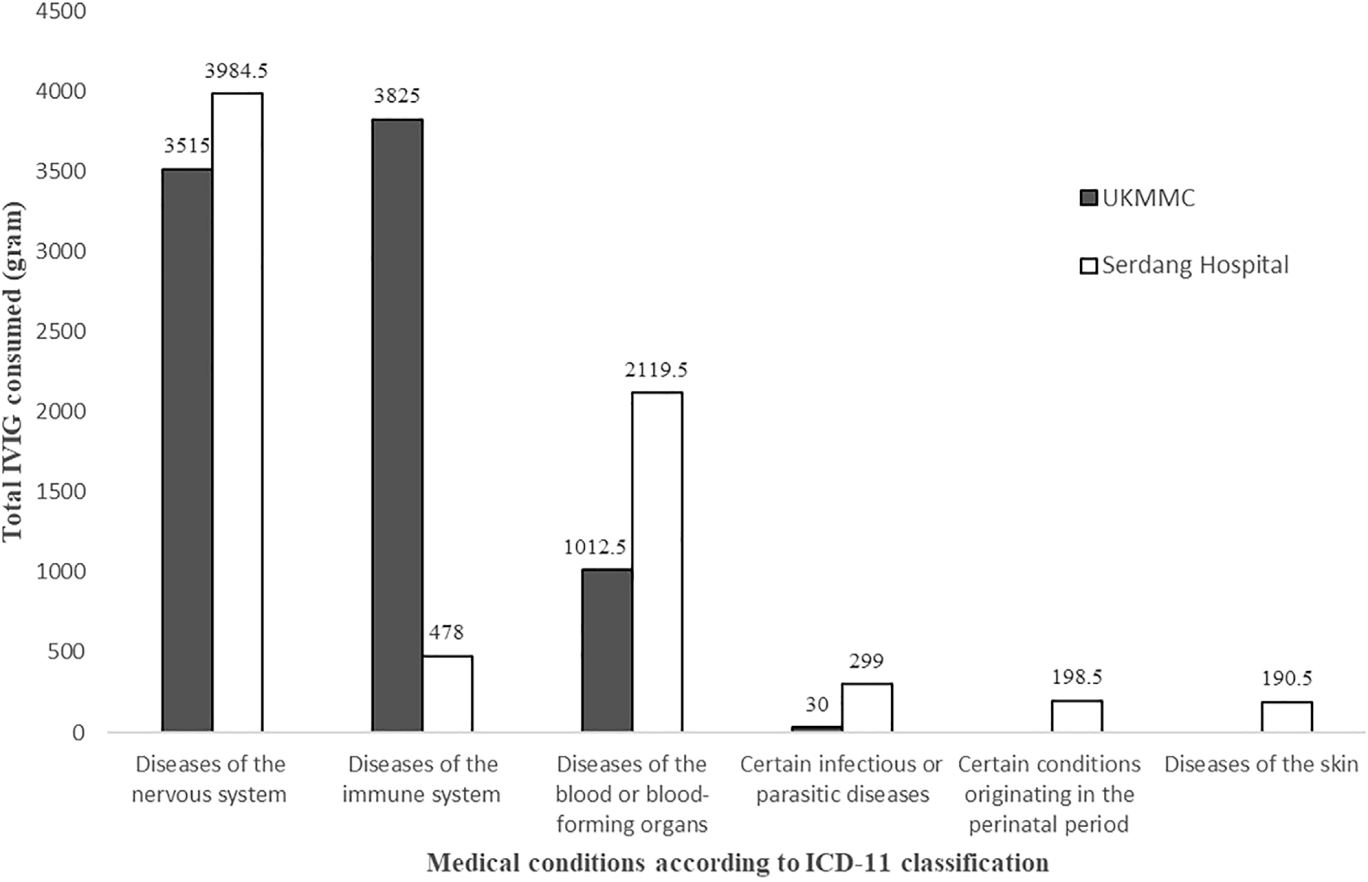
Total IVIG consumed according to medical conditions as per International Classification of Diseases 11th Revision (ICD-11) for Mortality and Morbidity Statistics (Version: 05/2021) classification.

The highest usage of IVIG was for neurological conditions (7,499.5 g, 47.9%), followed by immunological conditions (4,303 g, 27.5%) and hematological conditions (3,132 g, 20%) (Figure 1). Neurological conditions included Guillain-Barre syndrome (GBS), myasthenia gravis, autoimmune encephalitis, and chronic inflammatory demyelinating polyneuropathy (CIDP); immunological conditions included primary immunodeficiencies (PI), systemic lupus erythematosus (SLE), Kawasaki disease, and prevention of acute graft-versus-host disease (GVHD) whereas immune thrombocytopenia (ITP) and autoimmune hemolytic anemia (AIHA) were among the hematological conditions.

### Licensed and Off-Label Indication of Intravenous Immunoglobulin

IVIG was prescribed for 22 different indications. The number of prescriptions with FDA licensed and off-label indications were 148 (42.5%) and 200 (57.5%), respectively. The total cost of IVIG used for licensed indications was MYR 1,377,216 (37.24%) while a higher amount was spent on off-labeled indications (MYR 2,321,313, 62.76%). The highest proportion of prescriptions was written for PI (*n* = 82, 23.6%), followed by ABO hemolytic disease of the newborn (*n* = 46, 13.2%), and ITP (*n* = 42, 12.1%). The full description of IVIG utilization pattern along with the level of evidence and strength of recommendation is presented in Table 2.

**TABLE 2:** Utilization pattern of IVIG according to the level of evidence, the strength of recommendation, and beneficial category of treatment.

Disease Type	Level of Evidence and Strength of recommendation^a^	No of Prescriptions (%) (*n* = 348)	Total Grams of IVIG Used (%) [Cost]
**Licensed indication, definitely beneficial**			
Immune thrombocytopenia (ITP)	Ia (A)	42 (12.1)	3,123 (21) [MYR 737,934]
Kawasaki disease	Ia (A)	21 (6)	521 (3.3) [MYR 123,107]
Chronic inflammatory demyelinating polyneuropathy (CIDP)	Ia (A)	1 (0.3)	60 (0.4) [MYR 14,177]
Primary immunodeficiencies (PI)	IIb (B)	82 (23.6)	1937 (12.4) [MYR 457,694]
**Licensed indication, may provide benefit**			
Prevention of acute graft-versus-host disease (GVHD) post bone marrow transplant	Ib (A)	2 (0.6)	187.5 (1.2) [MYR 44,304]
**Total prescriptions with licensed indications [Total cost]**		**148 (42.5)**	5828.5 (37.2) [MYR 1,377,216]*
**Off-label, definitely beneficial**			
Cytomegalovirus pneumonitis	Ib (A)	1 (0.3)	12 (0.08) [MYR 2,835]
Guillain-Barre syndrome (GBS)	Ib (B)	38 (10.9)	4,554.5 (29.1) [MYR 1,076,183]
**Off-label, probably beneficial**			
Myasthenia gravis in crisis or before surgery	Ib (B)	17 (4.9)	2,392.5 (15.3) [MYR 565,324]
Toxic epidermal necrolysis	IIa (B)	1 (0.3)	165 (1.1) [MYR 38,988]
**Off-label, may provide benefit**			
Autoimmune encephalitis	III (C)	5 (1.4)	492.5 (3.1) [MYR 116,373]
Acute myocarditis	III (C)	1 (0.3)	24 (0.2) [MYR 5,671]
Autoimmune hemolytic anemia	III (D)	1 (0.3)	9 (0.06) [MYR 2,127]
ABO hemolytic disease of the newborn	III (D)	46 (13.2)	133.5 (0.9) [MYR 31,545]
Systemic lupus erythematosus (SLE)	III (D)	16 (4.6)	1657.5 (10.6) [MYR 391,651]
Varicella-zoster post-exposure prophylaxis^b^	IV (D)^b^	5 (1.4)	13.5 (0.09) [MYR 3,190]
**Off-label, unlikely to provide benefit**			
Neonatal sepsis^c^	Ia (A)^c^	14 (4)	53 (0.3) [MYR 12,523]
Post-operative sepsis in children^c^	III (C)^c^	22 (6.3)	102 (0.7) [MYR 24,102]
Sepsis in children^c^	NA	26 (7.5)	182 (1.2) [MYR 43,005]
**Off-label, not rated**			
Varicella-zoster treatment	NA	1 (0.3)	12 (0.08) [MYR 2,835]
Measles post-exposure prophylaxis	NA	2 (0.6)	6 (0.04) [MYR 1,418]
Measles treatment	NA	1 (0.3)	3 (0.02) [MYR 709]
Neonatal jaundice secondary to causes other than ABO incompatibility	NA	3 (0.9)	12 (0.9) [MYR 2,835]
**Total prescriptions with off-label indications [Total cost]**		**200 (57.5)**	9,824 (62.8) [MYR 2,321,313]*

MYR, malaysian ringgit, * The mean cost of IVIG, used in this study is MYR236.29 (57 USD) per Gram.

a Level of evidence, the strength of recommendation, and ordinal category are according to Perez et al. (Perez et al., 2017), unless stated otherwise.

b Level of evidence is according to Prairie Collaborative Immune Globulin Utilization Management Framework Project (Prairie Collaborative, 2018).

c Ordinal category is according to the latest meta-analysis by Ohlsson and Lacy (Ohlsson and Lacy, 2020).

Among the 148 prescriptions with licensed indications, the most common were for PI (*n* = 82, 55.4%), ITP (*n* = 42, 28.4%), and Kawasaki disease (*n* = 21, 14.2%). Of the 200 prescriptions for off-labelled indications, the most common were for ABO hemolytic disease of the newborn (*n* = 46, 23%), GBS (*n* = 38, 19%), and sepsis (*n* = 26, 13%). For off-label indications, 39 (19.5%) were categorized as “definitely beneficial”, 18 (9%) as “probably beneficial”, 74 (37%) categorized as “may provide benefits”, 62 (31%) as “unlikely to provide benefit”, and the intended use were not rated for 7 (3.5%) prescriptions.

Age, sex, ethnicity, and ward setting were investigated as potential factors affecting unlicensed use of IVIG and the results of the logistic regression analysis are shown in Table 3. Patients admitted to the critical care units were more likely to receive IVIG for an unlicensed indication (OR: 11.11, 95% CI: 5.60–22.05, *p* < 0.001). The odds of receiving IVIG for off-label indications increased by 2.2% as patients get older by a year (OR: 1.022, 95% CI: 1.007–1.036, *p* < 0.001).

**TABLE 3:** Predictors for receiving IVIG for an unlicensed indication and inappropriate dose of IVIG.

Predictors	Odds Ratio	95% confidence Interval		*p*-value
		**Lower**	**Upper**	
***Predictors for receiving IVIG for an unlicensed indication***				
**Sex**				
Female	1.186	0.653	2.153	0.575
Male (Reference)				
**Ethnicity**				0.97
Chinese	1.109	0.461	2.669	0.817
Indians	0	-	-	0.999
Others	0.700	0.135	3.625	0.671
Bumiputera (Reference)				
**Age**	1.022	1.007	1.036	0.003^a^
**Ward setting**				<0.001^a^
Critical care units	11.107	5.595	22.048	<0.001^a^
Daycare units	0	-	-	0.996
General wards (Reference)				
***Predictors for receiving inappropriate dosing of IVIG***				
**Sex**				
Female	0.738	0.392	1.390	0.347
Male (Reference)				
**Ethnicity**				0.353
Chinese	1.894	0.648	5.539	0.243
Indians	2.563	0.481	13.663	0.270
Others	2.647	0.400	17.495	0.312
Bumiputera (Reference)				
**Age**	0.931	0.894	0.970	0.001^a^
**Ward setting**				<0.001^a^
Critical care units	10.154	3.810	27.063	<0.001^a^
Daycare units	0	-	-	0.997
General wards (Reference)				

a Statistically significant results, *p* < 0.05.

### Appropriateness of Prescribed Dose

The prescribed dose used in this study compared with the recommended dose from the clinical practice guidelines of IVIG used in three countries (Prairie Collaborative, 2018; Jurisdictional Blood Committee, 2012; Department of Health, 2011; NHS England Immunoglobulin Policy Working Group, 2019; Department of Health, 2008) and product insert of the brands applied in the hospitals (Instituto Grifols, 2017; Behring, 2016) (Supplementary Table S2). Besides prescriptions for neonates, the prescribed doses were rounded to the nearest vial size available in the respective hospitals to avoid wastage (Department of Health, 2011; Stump et al., 2017). Most of the prescriptions (*n* = 275, 79.0%) had appropriate dosing while 19.54% (*n* = 68) lacked recommendation of the prescribed dose, which comprises seven indications: neonatal sepsis, sepsis in children, post-operative sepsis, acute myocarditis, treatment of varicella-zoster, treatment of measles, and neonatal jaundice (excluding ABO hemolytic disease of the newborn). Only five (1.44%) prescriptions were prescribed at a different dose from the recommendation and they were for the prevention of graft-versus-host disease, cytomegalovirus pneumonitis, and post-exposure prophylaxis for measles.

For the logistic regression analysis, the inappropriate dose included prescriptions without dose recommendation and those administered doses different from the recommendation. As shown in Table 3, patients admitted to the critical care units were more likely to receive an inappropriate dose of IVIG (OR: 10.15, 95% CI: 3.81–27.06, *p* < 0.001). Additionally, the odds of receiving an inappropriate dose of IVIG decreased by 7% as patients get older by a year (OR: 0.93, 95% CI: 0.894–0.970, *p* = 0.001).

## Discussion

The data described in this study reflect the patterns of IVIG used in two government tertiary care hospitals in Malaysia. A total of MYR 3.7 million (885,220 USD) was spent in the two hospitals for IVIG used for treating various conditions. The array of indications reflects the growing list of indications for which IVIG is currently approved. The leading groups of conditions using a high amount of IVIG were diseases of the nervous, immune, and circulatory systems. These findings are consistent with the reports from other studies (Pendergrast et al., 2005; Constantine et al., 2007; Lin et al., 2007).

The findings revealed that more than half of the prescriptions for IVIG (57.5%) were for FDA off-label indications. Similar prescribing pattern studies have been conducted in several countries and the prevalence of prescriptions with off-label indications of IVIG ranged from 22.7 to 70.3% (Rezaie et al., 2019; Tonkovic and Rutishauser, 2014; Toh et al., 2014; Ruiz-Antorán et al., 2010; Constantine et al., 2007; Pendergrast et al., 2005; El Ajez et al., 2019) (Supplementary Table S3). Likewise, the prevalence of patients being prescribed with IVIG for an off-label indication ranged from 23.2 to 81.5% (Chen et al., 2000; Pendergrast et al., 2005; Dawoud et al., 2012; Wu et al., 2013; Fakhari et al., 2018; Rezaie et al., 2019). The wide range could be due to different categorization or interpretation of off-labels, study periods, or duration of data collection, as licensing of an indication may change over time with the availability of more studies supporting its use. The prevalence and common off-label uses of IVIG that were reported in other studies is tabulated in Supplementary Table S3.

Prescriptions written for PI were the most frequent licensed indications in this study. This is expected as these patients are required to receive life-long IVIG at replacement doses of 0.3–0.8 g/kg, every three to 4 weeks. Moreover, IVIG is the first line and the only treatment option for PI, and its administration is generally recommended to maintain serum IgG levels above 500 mg/dl (Behring, 2016; Perez et al., 2017). Nevertheless, the total usage of IVIG per year for PI patients who were on regular three to four weekly doses was lower in this study compared to patients with GBS, ITP, and myasthenia gravis. These latter conditions require a one-off and a single course of high doses immunoglobulin of up to 2 g/kg for its immunomodulatory and anti-inflammatory effects (Behring, 2016; Perez et al., 2017).

IVIG has become an effective standard of care treatment for many off-label indications that are supported by many good clinical evidence (Perez et al., 2017). For instance, the use of IVIG in GBS and myasthenia gravis in crisis or before surgery is supported since both have a level of evidence and strength of recommendation of Ib (B) (Perez et al., 2017). The use of IVIG in GBS and myasthenia gravis have always been compared to the use of plasma exchange. In GBS, IVIG is labeled as ‘definite beneficial’ and its usage is supported by two large randomized controlled trials showing comparable efficacies to plasma exchange (van der Meché et al., 1992; Plasma Exchange/Sandoglobulin Guillain-Barre Syndrome Trial Group, 1997) and it is a well-recognized indication in many countries (Jurisdictional Blood Committee, 2012; European Medicines Agency, 2018; Prairie Collaborative, 2018; NHS England Immunoglobulin Policy Working Group, 2019). In myasthenic crises, IVIG was considered as a ‘possible benefit’ as its use is supported by two small randomized controlled trials (Gajdos et al., 1997; Barth et al., 2017). A recent meta-analysis indicated a higher response rate in plasma exchange compared to IVIG in patients with acute myasthenia gravis and those undergoing thymectomy (Ipe et al., 2021). In both conditions, IVIG is reported to be used more frequently compared to plasma exchange as it is more easily available in hospitals (Barth et al., 2017; Perez et al., 2017).

In this study, off-label indications where IVIG was unlikely to provide benefits comprised of neonatal sepsis, post-operative sepsis in children, and sepsis in children (Ohlsson and Lacy, 2020; Weiss et al., 2020). The reason for the high usage in these conditions could be due to the “desperate use” of IVIG, whereby a clinician tries to rescue a seriously-ill patient who has limited available treatment options despite the low benefit of the intervention compared to treatment costs (Pendergrast et al., 2005). In these circumstances, IVIG could be an appealing treatment option as it is relatively well-tolerated, despite insufficient evidence to support its use (Pendergrast et al., 2005; Perez et al., 2017).

The factors associated with the use of IVIG for off-label indications in this study were increasing age and those admitted to the critical care units. These results corroborate the findings by (Fakhari et al., 2018), whereby older age patients were more likely to receive IVIG for an off-labeled indication. Noticeably, the studies of IVIG utilization conducted for pediatric usage were mostly for FDA-licensed indications (Wu et al., 2013; Aydin and Tanır, 2017; El Ajez et al., 2019). It appears that IVIG is more likely to be tested out for new indications in adult diseases. In a critical care setting, IVIG may be used as the final therapeutic option in critically ill patients. Therefore, physicians are likely to prescribe IVIG even when the evidence to support its use is insufficient (Pendergrast et al., 2005). This observation was supported by a retrospective evaluation of IVIG use in medical ICU, whereby the investigators demonstrated that, of the six million US dollars spent over the 8 years for IVIG use, one-third of the cost was used in indications that have low-quality of evidence (Torbic et al., 2021). This could explain the high usage of IVIG for an off-label indication in critical care units.

The identification of predictors for use of IVIG in off-label indications is important when performing inventory rationing, especially where the supply of IVIG is limited. Such a situation could arise due to various reasons, such as product recall concerning quality issues, lack of raw materials, increase in demand, or a sudden halt in production (Shukar et al., 2021). A recent threat to shortages of IVIG supply occurred during the ongoing COVID-19 pandemic characterized by an increase in demand but a decrease in plasma donation, thereby resulting in limited availability of starting material (Hartmann and Klein, 2020). These factors should be considered the focus area when planning appropriate intervention to reduce the usage of IVIG for off-label indications. Justification of IVIG use needs to be considered on a case basis and prioritizing its supply to the patients who needed it most.

In this study, the IVIG doses prescribed were appropriate for most prescriptions. The majority of prescribed doses were rounded to the nearest vial size available to avoid wastage, as recommended by many guidelines (Department of Health, 2011; Stump et al., 2017). In cases lacking in dose recommendation or doses that differed from the recommendation, the doses could likely be obtained from other guidelines or case studies. For instance, the dose of 0.4 g/kg was available in the Centers for Disease Control and Prevention (CDC) guidelines in the case of post-exposure prophylaxis of measles (Centers for Disease Control and Prevention, 20132013). Factors associated with inappropriate dosing of IVIG were negatively correlated with age and those admitted to the critical care units. From the existing literature, no information was found about the factors related to inappropriate dosing of IVIG. The lack of research in children and critically-ill patients could be a possible reason for the lack of dosing information. Therefore, closer monitoring of treatment efficacy and adverse drug reaction is required in these groups of patients.

IVIG is generally considered as well-tolerated with the majority of adverse events reported to be infusion-related reactions (Guo et al., 2018). Moreover, these infusion-related reactions have been significantly reduced in the past 2 decades due to the improved manufacturing processes (Wood et al., 2007) and refined administration protocol (Aghamohammadi et al., 2004). In the literature, the reported rates of adverse drug reactions differed between studies and ranged from 3.8 to 6.1% of total administrations (Toh et al., 2014; Güngör and Yarali, 2020). In this study, no adverse drug reactions were documented in the medical records. This might be attributed to the slow infusion rates given as per administration protocol and the concurrent medication such as paracetamol, aspirin, and steroids that was given for the underlying medical condition might have masked the adverse reactions.

Given the high usage and cost of IVIG for off-label indications, specific guidelines for the clinical use of IVIG and an approval system for IVIG prescription are required. The health systems in several countries have taken the necessary step by implementing evidence-based guidelines with priority protocols for IVIG use (Boulis et al., 2002; Department of Health, 2008; Jurisdictional Blood Committee, 2012; Prairie Collaborative, 2018). A reduction of off-label uses of IVIG was observed after the implementation of these strategies (Constantine et al., 2007). Optimization of IVIG usage through establishing priorities is vital, especially during shortages. Such a procedure will protect the supply for those with utmost clinical need, where no alternative treatments are available, such as for the treatment of patients with PI (Meyts et al., 2020; Nordin et al., 2021). In 2018, an advocacy white paper entitled “A hidden health threat–Expert Recommendations for Better Management of Primary Immunodeficiency (PID) in Malaysia” developed by the Malaysian Patient Organisation for Primary Immunodeficiencies (MyPOPI) and Malaysian Society of Allergy and Immunology (MSAI) was presented to the Ministry of Health Malaysia. One of the highlighted pleas was for the stakeholders to ensure an uninterrupted supply of immunoglobulin replacement therapy for all PI patients (Paediatrics, 2021). This would further support the call for the development of a proper guideline of IVIG use in the country. Apart from the guidelines, the use of prior authorization or IVIG request form (Constantine et al., 2007; Tonkovic and Rutishauser, 2014) and IVIG stewardship program (Rocchio et al., 2017) are other ways of managing IVIG use. These strategies have been shown to decrease the inappropriate use of IVIG (Constantine et al., 2007; Rocchio et al., 2017). Recommendation for future work include a national survey evaluating the prescribing pattern of IVIG, as it would give better representation of its utilization in Malaysia. This is necessary for the development of national guideline on IVIG use. A steering committee consisting of experts from related fields would then establish a list of prioritization to ensure judicious use of IVIG in hospitals.

The limitations of this study are well-acknowledged. Only two public hospitals were involved in the data collection, thus the findings may not be representative of all hospitals in Malaysia, especially in the private setting. In addition, the assessment of evidence level was solely based on the most possible indication targeted by IVIG treatment and the disease complexity and severity were not considered. Furthermore, this study was conducted during the pre-COVID-19 period, hence the use in COVID-19 related illnesses was not captured. Nonetheless, this study provides useful insights on IVIG utilization patterns in hospitals, for which the data could be used for developing suitable approaches to optimize IVIG use in Malaysia.

## Conclusion

This study characterized the prescribing pattern of IVIG in two tertiary care hospitals in Malaysia. The two hospitals spent a total of MYR 3.7 million (885,220 USD) for IVIG used in various medical conditions. A high percentage of IVIG usage for off-label indications that lacked strong clinical evidence was observed. Most of the prescribed IVIG doses were appropriate while those with inappropriate doses were for indications lacking adequate clinical evidence. Admission to the critical care units and older patients were identified as risk factors for inappropriate use of IVIG, whereas admission to the critical units and younger patients were the risk factors for receiving inappropriate doses of IVIG. This group of patients requires more attention because they are likely to contribute to irrational use of IVIG. This study advocates the need for the development and implementation of evidence-based clinical guidelines with prioritization protocol to ensure rational use of IVIG in Malaysia. More research is also needed to establish therapeutic benefit that is lacking for several indications.

## Data Availability

The raw data supporting the conclusions of this article will be made available by the authors, without undue reservation.
