# Supplementary material for: Prescribing Practices of Intravenous Immunoglobulin in Tertiary Care Hospitals in Malaysia: A Need for a National Guideline for Immunoglobulin Use
Source: Front Pharmacol. 2022 Jun 9;13:879287. doi: 10.3389/fphar.2022.879287 (PMC9218597; doi:10.3389/fphar.2022.879287)
Supplement: Supplementary file 1 [file Table1.DOCX]

**Table 1.** Summarized data of included prescriptions (n = 348)

| **Parameter** |  |
| --- | --- |
| Sex, n (%)  Male  Female | 184 (52.9)  164 (47.1) |
| Median age (IQR; min, max)  Age class, n (%)  Preterm neonates (<37-week PMA up to 1 month)  Term neonates (≥37-week PMA up to 1 month)  Infants (1 month up to 2 years)  Children (2 years up to 12 years)  Adolescents (12 years to 18 years)  Adult (≥18 years) | 5.08 (2 months - 46 years; 1 day, 76 years)  21 (6.0)  55 (15.8)  61 (17.5)  57 (16.4)  20 (5.7)  134 (38.5) |
| Ethnic group, n (%)  Bumiputera^a^  Chinese  Indians  Others^b^ | 264 (75.9)  68 (19.5)  8 (2.3)  8 (2.3) |
| Ward settings, n (%)  Critical care units  Daycare units  General wards | 155 (44.5)  71 (20.4)  122 (35.1) |
| Total grams of IVIG used, grams  2.5g/50mL bottle, grams  3.0g/50mL bottle, grams | 15,652.5  10,537.5  5115.0 |
| Total cost^c^ | MYR 3,698,529 (885,218 USD) |

PMA = post-menstrual age, IVIG = intravenous immunoglobulin, MYR = Malaysian Ringgit

^a^ Bumiputera includes Malays (n = 260) and the natives from Sabah (n = 3) and Sarawak (n = 1).

^b^ Others include non-Malaysian citizens.

^c^ Total cost calculation was based on the mean cost of IVIG of MYR236.29 (57 USD) per gram.
