# Supplementary material for: Prescribing Practices of Intravenous Immunoglobulin in Tertiary Care Hospitals in Malaysia: A Need for a National Guideline for Immunoglobulin Use
Source: Front Pharmacol. 2022 Jun 9;13:879287. doi: 10.3389/fphar.2022.879287 (PMC9218597; doi:10.3389/fphar.2022.879287)
Supplement: Supplementary file 2 [file Table2.DOCX]

**Table 2.** Utilization pattern of IVIG according to the level of evidence, the strength of recommendation, and beneficial category of treatment

| **Disease type** | **Level of evidence and strength of recommendation^a^** | **No of prescriptions (%)**  **(n = 348)** | **Total grams of IVIG used (%) [cost]** |
| --- | --- | --- | --- |
| ***Licensed indication, definitely beneficial*** |  |  |  |
| Immune thrombocytopenia (ITP) | Ia (A) | 42 (12.1) | 3123 (21) [MYR 737,934] |
| Kawasaki disease | Ia (A) | 21 (6) | 521 (3.3) [MYR 123,107] |
| Chronic inflammatory demyelinating polyneuropathy (CIDP) | Ia (A) | 1 (0.3) | 60 (0.4) [MYR 14,177] |
| Primary immunodeficiency (PI) | IIb (B) | 82 (23.6) | 1937 (12.4) [MYR 457,694] |
| ***Licensed indication, may provide benefit*** |  |  |  |
| Prevention of acute graft-versus-host disease (GVHD) post bone marrow transplant | Ib (A) | 2 (0.6) | 187.5 (1.2) [MYR 44,304] |
| Total prescriptions with licensed indications [Total cost] |  | 148 (42.5) | 5828.5 (37.2) [MYR 1,377,216]* |
| ***Off-label, definitely beneficial*** |  |  |  |
| Cytomegalovirus pneumonitis | Ib (A) | 1 (0.3) | 12 (0.08) [MYR 2,835] |
| Guillain-Barre syndrome (GBS) | Ib (B) | 38 (10.9) | 4554.5 (29.1) [MYR 1,076,183] |
| ***Off-label, probably beneficial*** |  |  |  |
| Myasthenia gravis in crisis or before surgery | Ib (B) | 17 (4.9) | 2392.5 (15.3) [MYR 565,324] |
| Toxic epidermal necrolysis | IIa (B) | 1 (0.3) | 165 (1.1) [MYR 38,988] |
| ***Off-label, may provide benefit*** |  |  |  |
| Autoimmune encephalitis | III (C) | 5 (1.4) | 492.5 (3.1) [MYR 116,373] |
| Acute myocarditis | III (C) | 1 (0.3) | 24 (0.2) [MYR 5,671] |
| Autoimmune hemolytic anemia | III (D) | 1 (0.3) | 9 (0.06) [MYR 2,127] |
| ABO hemolytic disease of the newborn | III (D) | 46 (13.2) | 133.5 (0.9) [MYR 31,545] |
| Systemic lupus erythematosus (SLE) | III (D) | 16 (4.6) | 1657.5 (10.6) [MYR 391,651] |
| Varicella-zoster post-exposure prophylaxis^b^ | IV (D)^b^ | 5 (1.4) | 13.5 (0.09) [MYR 3,190] |
| ***Off-label, unlikely to provide benefit*** |  |  |  |
| Neonatal sepsis^c^ | Ia (A)^c^ | 14 (4) | 53 (0.3) [MYR 12,523] |
| Post-operative sepsis in children^c^ | III (C)^c^ | 22 (6.3) | 102 (0.7) [MYR 24,102] |
| Sepsis in children^c^ | NA | 26 (7.5) | 182 (1.2) [MYR 43,005] |
| ***Off-label, not rated*** |  |  |  |
| Varicella-zoster treatment | NA | 1 (0.3) | 12 (0.08) [MYR 2,835] |
| Measles post-exposure prophylaxis | NA | 2 (0.6) | 6 (0.04) [MYR 1,418] |
| Measles treatment | NA | 1 (0.3) | 3 (0.02) [MYR 709] |
| Neonatal jaundice secondary to causes other than ABO incompatibility | NA | 3 (0.9) | 12 (0.9) [MYR 2,835] |
| Total prescriptions with off-label indications [Total cost] |  | 200 (57.5) | 9824 (62.8) [MYR 2,321,313]* |

MYR = Malaysian Ringgit, * The mean cost of IVIG used in this study is MYR236.29 (57 USD) per gram.

^a^ Level of evidence, the strength of recommendation, and ordinal category are according to Perez et al (3), unless stated otherwise.

^b^ Level of evidence is according to Prairie Collaborative Immune Globulin Utilization Management Framework Project (5).

^c^ Ordinal category is according to the latest meta-analysis by Ohlsson and Lacy (53).
