# Supplementary material for: Prescribing Practices of Intravenous Immunoglobulin in Tertiary Care Hospitals in Malaysia: A Need for a National Guideline for Immunoglobulin Use
Source: Front Pharmacol. 2022 Jun 9;13:879287. doi: 10.3389/fphar.2022.879287 (PMC9218597; doi:10.3389/fphar.2022.879287)
Supplement: Supplementary file 3 [file Table3.DOCX]

**Table 3.** Predictors for receiving IVIG for an unlicensed indication and inappropriate dose of IVIG

| **Predictors** | **Odds ratio** | **95% Confidence interval** | | **p-value** |
| --- | --- | --- | --- | --- |
|  |  | **Lower** | **Upper** |  |
| ***Predictors for receiving IVIG for an unlicensed indication*** | | | | |
| **Sex** |  |  |  |  |
| Female | 1.186 | 0.653 | 2.153 | 0.575 |
| Male (reference) |  |  |  |  |
| **Ethnicity** |  |  |  | 0.97 |
| Chinese | 1.109 | 0.461 | 2.669 | 0.817 |
| Indians | 0 | - | - | 0.999 |
| Others | 0.700 | 0.135 | 3.625 | 0.671 |
| Bumiputera (reference) |  |  |  |  |
| **Age** | 1.022 | 1.007 | 1.036 | 0.003* |
| **Ward setting** |  |  |  | < 0.001* |
| Critical care units | 11.107 | 5.595 | 22.048 | < 0.001* |
| Daycare units | 0 | - | - | 0.996 |
| General wards (reference) |  |  |  |  |
| ***Predictors for receiving inappropriate dosing of IVIG*** | | | | |
| **Sex** |  |  |  |  |
| Female | 0.738 | 0.392 | 1.390 | 0.347 |
| Male (reference) |  |  |  |  |
| **Ethnicity** |  |  |  | 0.353 |
| Chinese | 1.894 | 0.648 | 5.539 | 0.243 |
| Indians | 2.563 | 0.481 | 13.663 | 0.270 |
| Others | 2.647 | 0.400 | 17.495 | 0.312 |
| Bumiputera (reference) |  |  |  |  |
| **Age** | 0.931 | 0.894 | 0.970 | 0.001* |
| **Ward setting** |  |  |  | < 0.001* |
| Critical care units | 10.154 | 3.810 | 27.063 | < 0.001* |
| Daycare units | 0 | - | - | 0.997 |
| General wards (reference) |  |  |  |  |

* Statistically significant results, p < 0.05
