# Supplementary material for: Prescribing Practices of Intravenous Immunoglobulin in Tertiary Care Hospitals in Malaysia: A Need for a National Guideline for Immunoglobulin Use
Source: Front Pharmacol. 2022 Jun 9;13:879287. doi: 10.3389/fphar.2022.879287 (PMC9218597; doi:10.3389/fphar.2022.879287)
Supplement: Supplementary file 4 [file Table4.DOCX]

**Supplementary material**

**Prescribing practices of intravenous immunoglobulin in tertiary care hospitals in Malaysia: A need for a national guideline for immunoglobulin use**

**Authors**

Jian Lynn Lee, Shamin Mohd Saffian, Mohd Makmor-Bakry, Farida Islahudin, Hamidah Alias, Adli Ali, Noraida Mohamed Shah

**Table S1. Definition of the categorization of evidence, strength of recommendation, and beneficial category of treatment**

| **Category** | **Definition** |
| --- | --- |
| **Evidence category** | |
| Ia | From meta-analysis of randomized controlled trials |
| Ib | From at least one randomized controlled trial |
| IIa | From at least one controlled trial without randomization |
| IIb | From at least one type of quasi-experimental study |
| III | From non-experimental descriptive studies such as comparative, correlation or case-control studies |
| IV | From expert committee reports or opinions or clinical experience of respected authorities or both |
| **Strength of recommendation** | |
| A | Based on category 1 evidence |
| B | Based on category II evidence or extrapolated from category I evidence |
| C | Based on category III evidence or extrapolated from category I or II evidence |
| D | Based on category IV evidence or extrapolated from category I, II or III evidence |
| NR | Not rated |
| **Ordinal category*** | |
| Definitely beneficial | |
| Probably beneficial | |
| May provide benefit | |
| Unlikely to provide benefit | |

* Consider evidence category and strength of recommendation in clinical decision-making regarding benefit of treatment with IVIG

**Table S2.** The prescribed dose in this study and the recommended dose based on criteria for clinical use of IVIG in three countries and the package leaflet of the IVIG products used in this study

| **Disease type** | **Prescribed dose in this study (g/kg) and its appropriateness**  **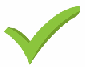 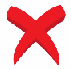 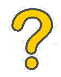** | **Recommended dose (g/kg)** | | | |
| --- | --- | --- | --- | --- | --- |
|  |  | **Criteria for the clinical use of intravenous immunoglobulin in Australia, Second edition**^6^ | **Criteria for the clinical use of immune globulin, First edition**^5^ | **Clinical Guidelines for Immunoglobulin Use, Second edition update**^7, 36, 37^ | **Product leaflets for Flebogamma^®^ 5% DIF**^34^ **and Intragam^®^ P 6%**^35^ |
| Licensed indication, definitely beneficial | | | | | |
| Immune thrombocytopenia | Children:  0.8g/kg OD for 1 day  (n = 16) *or*  0.8g/kg OD for 2 days  (n = 3) *or*  1g/kg OD for 1 day  (n = 2)  Adult:  0.4g/kg OD for 5 days  (n = 19) *or*  1g/kg OD for 2 days  (n = 2)  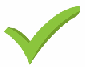 (n = 42) | Children:  1g/kg OD x 2 days, *or* 0.5g/kg as a single dose, repeated at 24 – 48 hours if response is inadequate.  Adult:  1 – 2g/kg as a single *or* divided dose over 2 – 5 days. | Children:  1g/kg OD x 2 days, *or* 0.8g/kg as a single dose, repeated within 48 hours if response is inadequate.  Adult:  1 - 2g/kg divided over 2 to 5 days. | Children:  0.8 – 1g/kg as a single infusion. A 2nd dose may be required after 24 – 48 hours if response is inadequate.  Adult:  1g/kg as a single infusion. A 2nd dose may be required after 24 – 48 hours if response is inadequate. | Flebogamma^®^ 5%  0.8 – 1g/kg on day 1, possibly repeated once within 3 days *or* 0.4g/kg for 2 – 5 days.  Intragam^®^ P 6%  Maximum cumulative dose of 2g/kg over 2 – 5 days. Adjustment of dose is empirical and should be based in the patient’s clinical state. |
| Kawasaki disease | 2g/kg OD for 1 day  (n = 21)  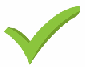 (n = 21) | 2g/kg in a single dose over 10 - 12 hours. Re-treatment with 2g/kg in a single dose may be given when there is ongoing inflammation. | 2g/kg in a single dose over 10 - 12 hours. One additional dose may be given if there is ongoing inflammation. | 2g/kg single dose, given over 10–12 hours; a second dose may be given if no response, or if relapse within 48h. | Flebogamma^®^ 5%  1.6 – 2g/kg in divided over 2 – 5 days in association with acetylsalicylic acid or 2g/kg in one dose in association with acetylsalicylic acid.  Intragam^®^ P 6%  1.6 - 2.0g/kg over 2 – 5 days *or* 2g/kg as a single dose wuth concomitant treatment with acetylsalicylic acid. |
| Chronic inflammatory demyelinating polyneuropathy | 0.2g/kg OD for 5 days  (n = 1)  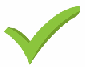 (n = 1) | Induction:  1 - 2g/kg in 2 to 5 divided doses.  Maintenance:  0.4–1g/kg, 2–6 weekly. The amount per dose should be titrated to the lowest dose necessary according to individual’s response. for 3 – 6 courses to see benefit. | 2g/kg adjusted body weight divided over 2 to 5 days, every 4 weeks. IVIG should be administered for 3 to 6 months to assess efficacy. | 2 doses of IVIg (2g/kg given over several days) 6 weeks apart; restarted at relapse and repeated using the ‘time to relapse’ as the interval between courses (i.e. if a patient relapses after 6 weeks, 2g/kg is given over several days every 6 weeks). | NA |
| Primary immunodeficiency | 0.4 – 0.7g/kg, every 3 – 4 weeks (n = 82)  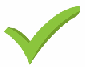 (n = 82) | 0.4g/kg every four weeks, modifying dose and schedule to achieve IgG trough level of at least the lower limit of the age-specific serum IgG reference range. | 0.4 to 0.6g/kg adjusted body weight IVIG every 4 weeks, modified to achieve an IgG trough level of at least the lower limit of the age-specific serum IgG reference range, or as needed to achieve clinical effectiveness. | 0.4–0.6g/kg/month; dose requirements may increase and should be based on clinical outcome. | Flebogamma^®^ 5%  Starting dose 0.4 – 0.8g/kg, then 0.2 – 0.8g/kg every 3 – 4 weeks to obtain IgG trough levels of at least 5 – 6 g/L.  Intragam^®^ P 6%  Starting dose is 0.4 – 0.6g/kg/month, then 0.2 – 0.6g/kg/month either as single dose or as 2 equal doses at fortnightly intervals. Adjustment of dose is empirical and should be based on patient’s clinical state and the pre-infusion IgG level. |
| Licensed indication, may provide benefit | | | | | |
| Prevention of acute graft-versus-host disease post in bone marrow transplant | 0.4g/kg OD for 1 day  (n = 1) *or*  1g/kg OD for 2 days  (n = 1)  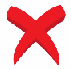 (n = 2) | NA | Not recommended | NA | Flebogamma^®^ 5%  0.5g/kg every week from day 7 up to 3 months after transplantation.  Intragam^®^ P 6%  Start dose at 0.5g/kg/week. |
| Off-label, definitely beneficial | | | | | |
| Cytomegalovirus pneumonitis | 1g/kg OD for 1 day  (n = 1)  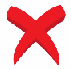 (n = 1) | NA | 0.4g/kg OD x 7 - 14 days. | 0.5g/kg OD x 5 days. | NA |
| Guillain-Barre syndrome | Children:  0.8g/kg OD for 2 days  (n = 1)  *or*  1g/kg OD for 2 days  (n = 2)  or  0.4g/kg OD for 5 days  (n = 5)  Adult:  0.4g/kg OD for 5 days  (n = 30)  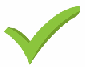 (n = 38) | 2g/kg in 2 to 5 divided doses. Approximately 10% of patients relapse, which may require a second treatment with IVIg. A second dose of IVIg must only be on the advice of and after assessment by a neurologist. | 2g/kg divided over 2 to 5 days. A second course of IVIG may be considered in patients with clearly demonstrated secondary deterioration, after assessment by a neurologist. | 2g/kg given over 5 days (shorter time frame not recommended because of potential fluid overload and autonomic problems). Second dose may be considered at 14 days for non- responsive or late deteriorating patients if IgG not increased from baseline by > 7g/L. | Flebogamma^®^ 5%  0.4g/kg for 5 days.  Intragam^®^ P 6%  0.4g/kg for 5 days. |
| Off-label, probably beneficial | | | | | |
| Myasthenia gravis in crisis or before surgery | 0.4g/kg OD for 5 days  (n = 17)  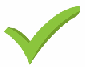 (n = 17) | Induction or before surgery, or during myasthenic crisis: 1–2g/kg in 2 to 5 divided doses.  Maintenance: 0.4–1g/kg, 4–6 weekly. | Induction, before surgery, or during myasthenic crisis: 1 to 2g/kg adjusted body weight divided over 2 to 5 days. Maintenance: 0.4 to 1g/kg adjusted body weight every 4 to 6 weeks. Once the patient’s condition has stabilized, consider titrating the dose and/or the treatment interval to the lowest dose necessary to maintain clinical effectiveness. | In acute exacerbation:  1g/kg in the first instance, only receiving a further 1g/kg if there is further deterioration or no response. | NA |
| Toxic epidermal necrolysis | 1g/kg OD for 3 days  (n = 1)  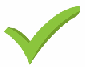 (n = 1) | 2g/kg, preferably as a single dose, *or* divided over three consecutive days. | One dose of 2g/kg, or 1g/kg/day for 3 consecutive days. IVIG should be initiated as early as possible, preferably within 24 hours of diagnosis. | 2g/kg, preferably as a single dose, *or* divided over three consecutive days. | NA |
| Off-label, may provide benefit | | | | | |
| Autoimmune encephalitis | 0.4g/kg OD for 5 days  (n = 4) *or*  1g/kg OD for 2 days  (n = 1)  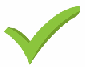 (n = 5) | NA | 2 g/kg divided over 2 to 5 days | NA | NA |
| Acute myocarditis | 1g/kg OD x 2 days  (n = 1)  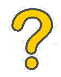 (n = 1) | NA | NA | NA | NA |
| Autoimmune hemolytic anemia | 1g/kg OD for 1 day  (n = 1)  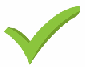 (n = 1) | Up to 2g/kg as a single *or* divided dose. | 1 to 2 g/kg divided over 2 to 5 days. | 1-2g/kg in two to five divided doses. This may be repeated on relapse and for a 2nd relapse. | NA |
| ABO hemolytic disease of the newborn | 0.5 g/kg OD for 1 day  (n = 36) *or*  0.8 g/kg OD for 1 day  (n = 1) *or*  1g/kg OD for 1 day  (n = 9)  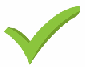 (n = 46) | NA | 1g/kg as a single dose | 0.5g/kg over 4 hours | NA |
| Systemic lupus erythematosus | 2g/kg divided over 2 to 5 days (n = 16)  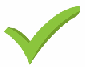 (n = 16) | Not recommended | 2g/kg divided over 2 to 5 days | NA | NA |
| Varicella zoster post-exposure prophylaxis | 0.4g/kg as a single dose  (n = 5)  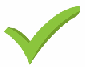 (n = 5) | NA | Only recommended when VZIG is contraindicated or unavailable,  0.4g/kg as a single dose. | Only recommended when VZIG is contraindicated or unavailable,  0.2g/kg as a single dose. | NA |
| Off-label, unlikely to provide benefit | | | | | |
| Neonatal sepsis | 0.5g/kg OD for 1 day  (n = 7)  0.5g/kg OD for 2 days  (n = 7)  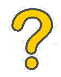 (n = 14) | Not recommended | Not recommended | Not recommended | NA |
| Post-operative sepsis in children | 0.5 - 1g/kg OD for 1 – 2 days (n = 22)  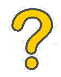 (n = 22) | NA | NA | NA | NA |
| Sepsis in children | 0.4 - 1g/kg OD x 1 – 4 days (n = 26)  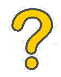 (n = 26) | NA | NA | Not recommended | NA |
| Off-label, not rated | | | | | |
| Varicella zoster treatment | 1g/kg as a single dose  (n = 1)  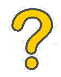 (n = 1) | NA | NA | NA | NA |
| Measles post-exposure prophylaxis | 0.4g/kg as a single dose  (n = 2)  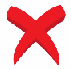 (n = 2) | NA | NA | 0.15g/kg of IVIg recommended ideally within 72 hours of exposure although can be given up to 6 days. | NA |
| Measles treatment | 0.4g/kg as a single dose  (n = 1)  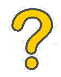 (n = 1) | NA | NA | NA | NA |
| Neonatal jaundice secondary to causes other than ABO incompatibility | 0.5kg/kg as a single dose  (n = 3)  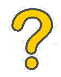 (n = 3) | NA | NA | NA | NA |


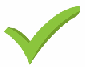
 (n): Number of prescriptions with dose within the recommended dose range.


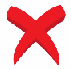
 (n): Number of prescriptions with dose lower or higher than the recommended dose range.


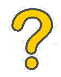
 (n): No dose recommendation.

**Table S3.** The prevalence and common off-label indications of IVIG in other utilization review studies

| **Author, year** | **Country** | **Study period** | **Percentages of off-label indications of IVIG (%)** | | **Common (top 5) off-label indications** |
| --- | --- | --- | --- | --- | --- |
|  |  |  | **Prescription** | **Patient** |  |
| Chen et al. 2000 (46) | Multicenter,  United States | 8 months | - | 52 | Prophylaxis of infection in solid organ transplantation, nonimmune thrombocytopenia, myasthenia gravis, BMT CMV prophylaxis in pediatric, hypogammaglobulinemia in high-risk neonates |
| Pendergrast et al. 2005 (43) | Multicenter, Canada | 6 years | 42.5 | 52.4 | Guillain-Barre syndrome, necrotizing fasciitis, autoimmune hemolytic anemia, myasthenia gravis, toxic epidermal necrolysis syndrome |
| Constantine et al. 2007 (42) | Multicenter, Canada | 6 months | 62.9 | - | Secondary immune deficiency conditions, myasthenia gravis, Guillain-Barre syndrome, rheumatoid arthritis, multiple sclerosis |
| Ruiz-Antorán et al. 2010 (28) | Multicenter, Spain | 3 months | 26 | 40 | Myasthenia gravis, dermatomyositis, multiple sclerosis, bullous dermatosis, transplanted organ complications |
| Dawoud et al. 2012 (9) | Single center, United Arab Emirates | 1 year | - | 63.4 | Sepsis, neonatal sepsis, thrombocytopenia, hemophagocytic lymphohistiocytosis, systemic lupus erythomatosus |
| Wu et al. 2013 (45) | Multicenter, Singapore (Pediatric) | 10 years | - | 23.2 | Secondary immunodeficiency in premature neonates, solid organ transplant, Guillain-Barre syndrome, encephalitis, post-infectious epilepsy |
| Toh et al. 2014 (26) | Single center, Malaysia (Pediatric) | 8 years | 47 | - | Neonatal sepsis, severe neonatal jaundice secondary to ABO incompabitibity, Guillain-Barre syndrome, perinatal acquired varicella infection (prophylaxis), neonatal autoimmune thrombcytopenia |
| Tonkovic & Rutishauser 2014 (11) | Multicenter,  United States | 4.5 years | 35.9 | - | Multiple sclerosis, myasthenia gravis with or without (acute) exacerbation, dermatomyositis, polymyositis, Guillain-Barre syndrome |
| Aydin & Tanir 2017 (55) | Single center, Turkey (Pediatric) | 4.5 years | - | 30.7 | Hemophagocytic lymphohistiocytosis, Steven-Johnson syndrome/ Toxic epidermal necrolysis, toxic shock syndrome, autoimmune encephalitis, invasive group A streptococci infection |
| Shemer et al. 2018 (10) | Single center,  Israel | 9 years | - | 56 | Myasthenia gravis, secondary immunodeficiency after immunosuppressive treatment, Guillain-Barre syndrome, multiple sclerosis, fetal alloimmunization |
| Fakhari et al. 2018 (39) | Single center,  Iran | 9 months | - | 47.5 | Guillain-Barre syndrome, in vitro fertilization, myasthenia gravis (acute exacerbation), postrenal transplantation, polymyositis (refractory) |
| El Ajez et al. 2019 (44) | Single center, Qatar  (Pediatric) | 1 year | 22.7 | - | Opsoclonus myoclonus, dermatomyositis, sepsis/ septic shock, acute disseminated encephalomyelitis, chylothorax |
| Rezaie et al. 2019 (8) | Single center, Iran | 10 months | 70.3 | 81.5 | Guillain-Barre syndrome, myasthenia gravis (acute exacerbation), dermatomyositis/ polymyositis (refractory), acquired hypogammaglobulinemia secondary to malignancy, |
| Gungor & Yaralı 2020 (61) | Single center, Turkey (Pediatric) | 2.5 years | - | 54.3 | Myocarditis, sepsis, Guillain-Barre syndrome, hemolytic anemia (ABO-RH incompatibility), autoimmune limbic encephalitis |
